# Activation of the S100A7/RAGE Pathway by IGF-1 Contributes to Angiogenesis in Breast Cancer

**DOI:** 10.3390/cancers13040621

**Published:** 2021-02-04

**Authors:** Maria Grazia Muoio, Marianna Talia, Rosamaria Lappano, Andrew H. Sims, Veronica Vella, Francesca Cirillo, Livia Manzella, Marika Giuliano, Marcello Maggiolini, Antonino Belfiore, Ernestina Marianna De Francesco

**Affiliations:** 1 Endocrinology, Department of Clinical and Experimental Medicine, University of Catania, Garibaldi-Nesima Hospital, 95122 Catania, Italy; mariagrazia.muoio@unict.it (M.G.M.); veronica.vella@unict.it (V.V.); marika.giuliano@phd.unict.it (M.G.); 2 Department of Pharmacy, Health and Nutritional Sciences, University of Calabria, 87036 Rende, Italy; marianna.talia@unical.it (M.T.); rosamaria.lappano@unical.it (R.L.); francesca.cirillo@unical.it (F.C.); marcello.maggiolini@unical.it (M.M.); 3 Applied Bioinformatics of Cancer, University of Edinburgh Cancer Research UK Centre, Institute of Genetics and Molecular Medicine, Crewe Road South, Edinburgh EH4 2XU, UK; andrew.sims@ed.ac.uk; 4 Center of Experimental Oncology and Hematology, A.O.U. Policlinico Vittorio Emanuele, 95122 Catania, Italy; manzella@unict.it; 5 Department of Clinical and Experimental Medicine, University of Catania, 95122 Catania, Italy

**Keywords:** IGF-1, IGF-1R, S100A7/psoriasin, RAGE, breast tumor microenvironment, tumor angiogenesis, STAT3, IGF-1R knock-out

## Abstract

**Simple Summary:**

Breast cancer mortality is increased in patients affected by metabolic disorders associated with dysregulation of the Insulin-like growth factor-1 (IGF-1) axis, like obesity and type-2 diabetes. Despite the oncogenic role of this complex signaling system is widely known, the clinical targeting of IGF-1 and its receptor (IGF-1R) has provided valuable benefit only on small sub-populations of cancer patients, thus suggesting that a further characterization of the biological effects of the IGF-1/IGF-1R pathway could pave the way for a better manipulation of this crucial signaling system at the clinical level. In this study, we have identified the protein S100A7 as novel molecular target of IGF-1 action in the breast tumor microenvironment, toward increased cancer-associated angiogenesis. Targeting the IGF-1/IGF-1R/S100A7 pathway may therefore represent a further useful approach for blocking disease progression in breast cancer patients with dysregulated IGF-1 signaling.

**Abstract:**

Background: Breast cancer (BC) mortality is increased among obese and diabetic patients. Both obesity and diabetes are associated with dysregulation of both the IGF-1R and the RAGE (Receptor for Advanced Glycation End Products) pathways, which contribute to complications of these disorders. The alarmin S100A7, signaling through the receptor RAGE, prompts angiogenesis, inflammation, and BC progression. Methods: We performed bioinformatic analysis of BC gene expression datasets from published studies. We then used Estrogen Receptor (ER)-positive BC cells, CRISPR-mediated IGF-1R KO BC cells, and isogenic S100A7-transduced BC cells to investigate the role of IGF-1/IGF-1R in the regulation of S100A7 expression and tumor angiogenesis. To this aim, we also used gene silencing and pharmacological inhibitors, and we performed gene expression and promoter studies, western blotting analysis, ChIP and ELISA assays, endothelial cell proliferation and tube formation assay. Results: S100A7 expression correlates with worse prognostic outcomes in human BCs. In BC cells, the IGF-1/IGF-1R signaling engages STAT3 activation and its recruitment to the S100A7 promoter toward S100A7 increase. In human vascular endothelial cells, S100A7 activates RAGE signaling and prompts angiogenic effects. Conclusions: In ER-positive BCs the IGF-1 dependent activation of the S100A7/RAGE signaling in adjacent endothelial cells may serve as a previously unidentified angiocrine effector. Targeting S100A7 may pave the way for a better control of BC, particularly in conditions of unopposed activation of the IGF-1/IGF-1R axis.

## 1. Introduction

The prognosis of breast cancer (BC) in patients with metabolic disorders such as obesity and type-2 diabetes (T2DM) is typically worse [1,2,3]. In this sub-population of BC patients an abnormal activation of the IGF/Insulin system is known to foster the acquisition of aggressive features through multiple mechanisms that entail both BC cells and the surrounding microenvironment [3,4,5]. In particular, IGF-1 binding to its receptor, namely IGF-1R, promotes malignant features not only by activating autocrine signaling in BC cells, but also by establishing paracrine communications that facilitate cell migration, invasion and new blood vessels formation [5,6,7]. On the basis of these observations, the identification of IGF-1/IGF1R-regulated molecular effectors, and the characterization of their role in affecting BC microenvironment may provide previously unappreciated targets of molecular intervention. The S100 calcium-binding protein A7 (S100A7), which belongs to the S100 family of proteins, is expressed in diverse tumors, including BC [8]. Although not generally expressed in normal mammary epithelium, S100A7 levels increase in the early stages of progression. Indeed, S100A7 is expressed in hyperplasia and atypical hyperplasia, and is prominently expressed in pre-invasive carcinoma in situ and pre-invasive ductal carcinoma in situ, thus suggesting a crucial role in the onset of the invasive phenotype (reviewed in [8]). S100A7 up-regulation, which is mainly observed during conditions of stress and inflammation, also occurs in response to hormones and growth factors, including estrogen and Epidermal Growth Factor (EGF) [9,10,11]. As the expression of S100A7 appears to be more abundant in ER-negative BCs, a number of studies have revealed a tumor-promoting role of S100A7 in ER-negative contexts, where it promotes cell proliferation, migration, survival, angiogenesis and metastasis [12,13,14,15]. On the other hand, only few controversial findings have been reported for ER-positive BCs [16,17,18]. When released in the extracellular milieu, S100A7 acts as a cytokine-like molecule binding to its receptor RAGE (Receptor for Advanced Glycation End Products), a transmembrane protein belonging to the immunoglobulin superfamily [19]. Previous studies have shown that RAGE signaling is aberrantly activated in conditions of de-regulation of the IGF/Insulin axis, like obesity and T2DM [20]. Furthermore, an activated S100A7/RAGE pathway has been shown to promote angiogenic effects and facilitate breast metastasis dissemination [12]. On the basis of these observations, we asked whether S100A7 may be engaged by IGF-1 and activate a dysfunctional BC microenvironment conducive to the acquisition of aggressive features. We found that S100A7 expression correlates with negative prognostic outcomes also in ER-positive BC patients. We established that the IGF-1/IGF-1R axis triggers STAT3-dependent transcriptional activation of S100A7, which acts as a paracrine mediator in the breast tumor microenvironment. We showed that the IGF1-dependent activation of the S100A7/RAGE signaling induces the proliferation of human vascular endothelial cells and their assembly into vessel-like structures. Overall, our findings indicate that the S100A7/RAGE pathway may be considered as a novel facilitator of the IGF-1 action in the stimulatory cross-talk between BC cell and the surrounding microenvironment.

## 2. Results

### 2.1. S100A7 Correlates with Negative Prognostic Features in Breast Cancer Patients

**Figure 1:**
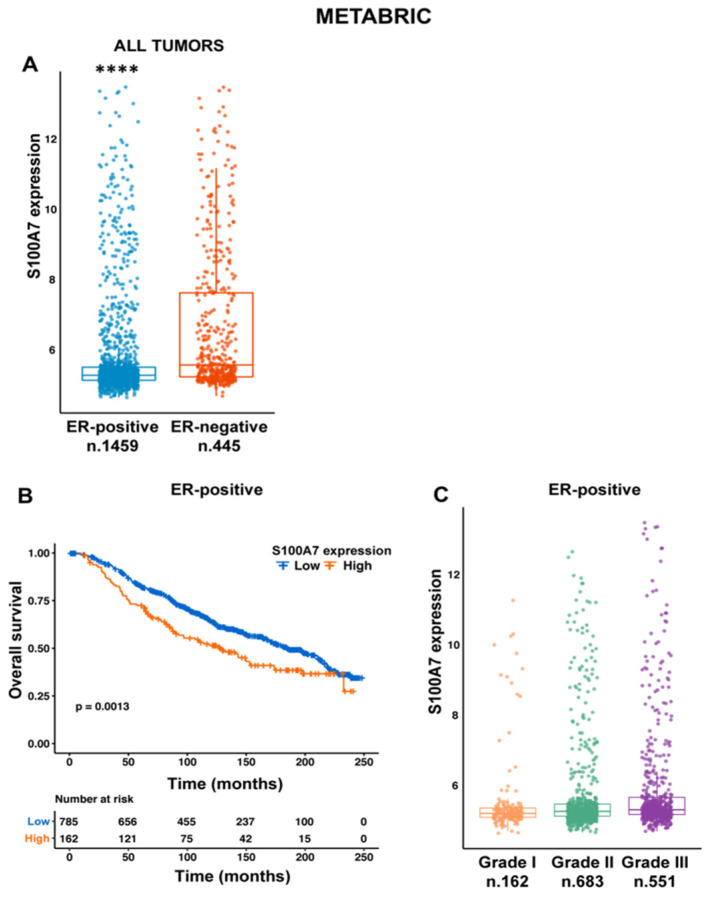
S100A7 correlates with adverse clinico-pathological features in ER-positive breast cancer patients. (**A**) Box plot depicting the differential expression of S100A7 in ER-positive and ER-negative BC patients as found in the METABRIC cohort; (****) *p* < 0.0001. (**B**) S100A7 expression correlates with a worse overall survival of ER-positive BC patients in the METABRIC cohort. (**C**) The expression levels of S100A7 were higher in ER-positive BC patients with more advanced tumor grade. (****) *p* < 0.0001; the number of patients of each group is indicated in the figure.

A number of studies have demonstrated the stimulatory role elicited by S100A7 in BC, particularly in the ER-negative histotypes, [12,13,14,15,21], whereas only few controversial studies have attempted to clarify the role of S100A7 in ER-positive breast tumors [16,17,18]. Therefore, we set out to verify whether S100A7 plays a stimulatory role in ER-positive BCs, which represent a sub-population of tumors more frequently associated with de-regulation of the IGF/Insulin pathway [5]. We began our investigation by interrogating the Molecular Taxonomy of Breast Cancer International Consortium (METABRIC) dataset, which collects gene expression data obtained using Illumina BeadChip and clinical parameters from 1904 BC patients. In line with previous data [22], we found that S100A7 is more abundantly expressed in ER-negative than in ER-positive BCs (Figure 1A). However, when we looked at the METABRIC ER-positive tumors, representing the largest sub-set of BC patients, we found that S100A7 expression correlates with a worse prognosis (Figure 1B), as well as with a higher tumor grade (Figure 1C). Together, these data suggest that S100A7 may represent a marker of disease aggressiveness in ER-positive BC patients.

### 2.2. IGF-1/IGF-1R Induces the Expression of S100A7

**Figure 2:**
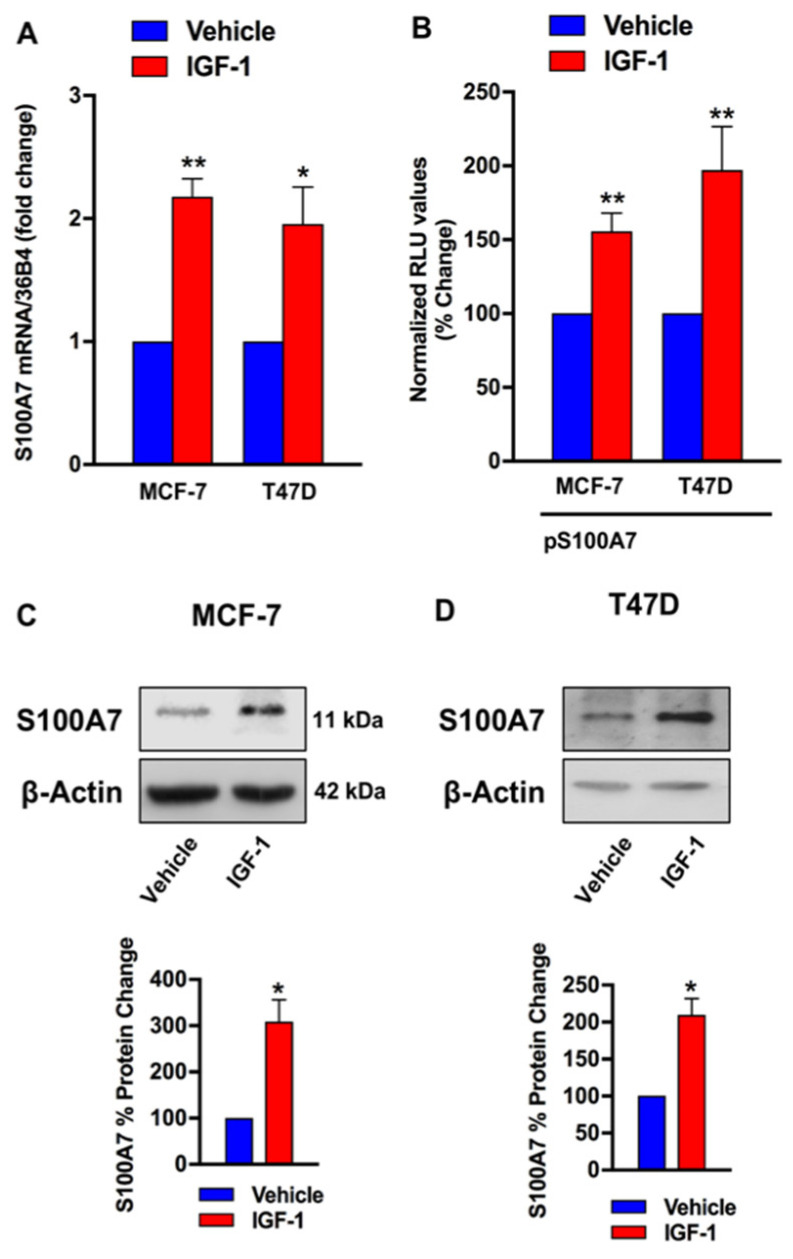
IGF-1 induces the expression of S100A7. (**A**) mRNA expression of S100A7 in MCF-7 and T47D cells treated vehicle or IGF-1 (10 nM, 48 h), as evaluated by qRT-PCR. Values were normalized to the 36B4 gene expression and shown as fold changes of mRNA expression induced by IGF-1 compared to cells treated with vehicle. Data are mean ± SEM of at least three independent experiments performed in duplicate. (**B**) Transactivation of a S100A7 (pS100A7) promoter construct observed in MCF-7 and T47D cells treated with IGF-1 (10 nM, 18 h). Luciferase activity was normalized to the internal transfection control. Results are expressed as the % change of normalized RLU values relative to vehicle-treated cells. Data shown are the mean ± SEM of at least two independent experiments performed in triplicate. (**C**,**D**) Representative immunoblots showing the increase of S100A7 protein expression in MCF-7 and T47D cells treated with IGF-1 (10 nM, 48 h). β-actin serves as loading control. Lower panels show densitometric analysis of the blots normalized to β-actin. Data shown are the mean ± SEM of at least two independent experiments. (*) *p* < 0.05; (**) *p* < 0.01.

**Figure 3:**
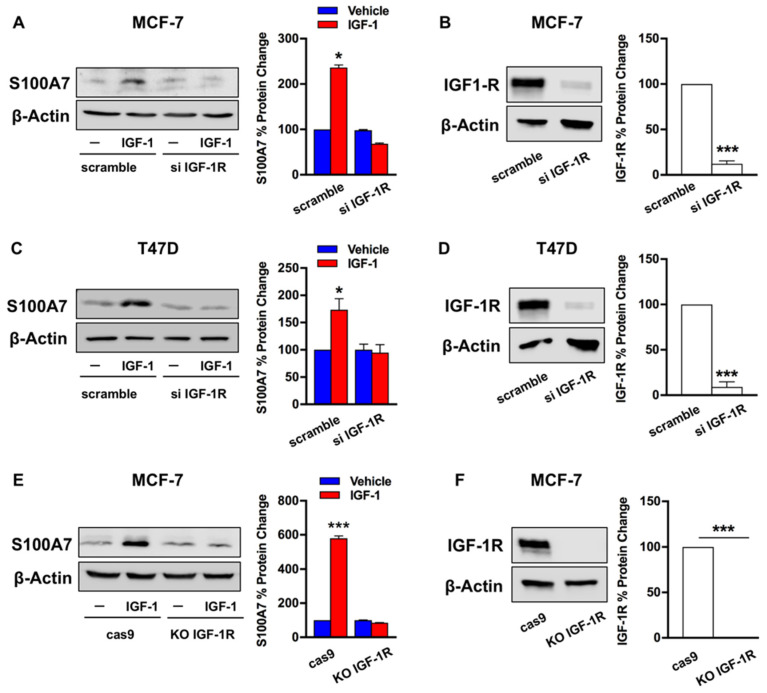
*IGF-1R* mediates the up-regulation of S100A7 expression induced by IGF-1. The upregulation of S100A7 protein expression observed in MCF-7 (**A**) and T47D (**C**) cells treated with vehicle (–) or IGF-1 (10 nM IGF-1, 48 h) was abrogated by silencing IGF-1R, as evidenced by western blotting. Representative immunoblots showing the efficiency of IGF-1R silencing in MCF-7 (**B**) and T47D cells (**D**). The up-regulation of S100A7 protein levels induced by IGF-1 (10 nM, 48 h) was prevented in MCF-7 cells knock out for IGF-1R (MCF-7 KO-IGF1R) compared to MCF-7 control cells (MCF-7 cas9), as indicated (**E**). Representative immunoblot showing the efficiency of IGF-1R knock-out (**F**). β-actin serves as loading control. Side panels show densitometric analysis of the blots normalized to β-actin. Data shown are the mean ± SEM of at least two independent experiments. (*) *p* < 0.05; (***) *p* < 0.001.

Based on the above data, we asked whether S100A7 may represent a target of the IGF-1 action in ER-positive breast tumors, which were modelled using two ER-positive breast cancer cells lines, MCF-7 and T47D respectively (Figure S1A,B). First, we found that IGF-1 up-regulates the mRNA expression of S100A7 (Figure 2A), as determined by qRT-PCR. Further corroborating these findings, we observed the transactivation of a S100A7 promoter construct (pS100A7) in both MCF-7 and T47D cells upon treatment with IGF-1 (Figure 2B). Accordingly, IGF-1 induced S100A7 protein expression in both MCF-7 and T47D cells (Figure 2C,D). Next, we asked whether IGF-1R is involved in the stimulatory effects elicited by IGF-1 on the expression of S100A7. By a gene silencing approach, we found that the up-regulation of S100A7 protein expression induced by IGF-1 is prevented knocking down IGF-1R in both MCF-7 and T47D cells (Figure 3A–D). Similarly, the increase of S100A7 protein levels observed upon stimulation with IGF-1 was no longer evident in MCF-7 cells engineered with a CRISPR-deletion of the IGF-1R gene (Figure 3E,F).

**Figure 4:**
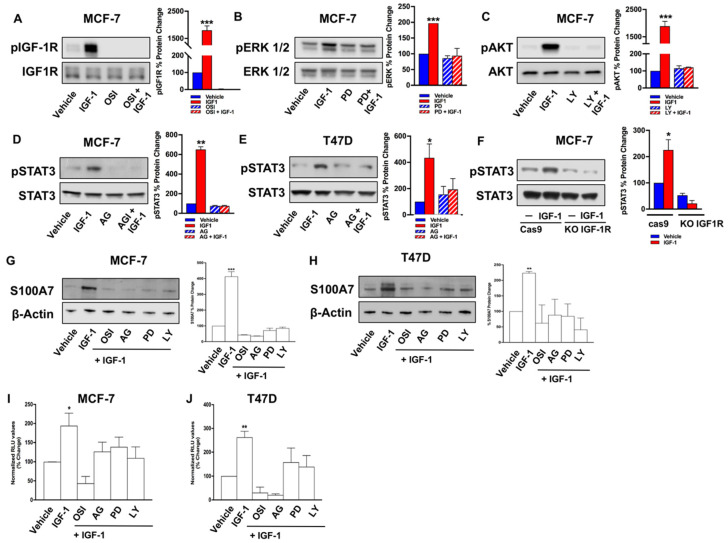
ERK1/2, AKT, and STAT3 signaling pathways are involved in the up-regulation of S100A7 expression induced by IGF-1. Phosphorylation of IGF-1R (**A**), ERK1/2 (**B**), and AKT (**C**) in MCF-7 cells treated with IGF-1 (10 nM, 24 h), alone and in combination with the IGF-1R inhibitor OSI-906 (OSI, 10 μM) (**A**), the MEK inhibitor PD0325901 (PD, 300 nM) (**B**), as well as the PI3K/AKT inhibitor LY294002 (LY, 10 μM) (**C**). In MCF-7 (**D**) and T47D (**E**) cells, the exposure to IGF-1 (10 nM, 24 h) induced the phosphorylation of STAT3 (Y705), which was prevented in the presence of the JAK/STAT inhibitor Tyrphostin AG490 (AG, 25 μM). (**F**) STAT3 phosphorylation (Y705) in response to IGF-1 (10 nM, 24 h) is absent in MCF-7 cells knock-out for IGF-1R (MCF-7 KO-IGF1R) via CRISPR genome editing, but present in MCF-7 control cells (MCF-7 cas9). The up-regulation of S100A7 protein expression was prevented in MCF-7 (**G**) and T47D (**H**) cells treated with IGF-1 (10 nM, 48 h), alone and in combination with OSI (OSI-906, 10 μM), AG (AG490, 25 μM), PD (PD0325901, 300 nM), and LY294002 (LY, 10 μM). β-actin, IGF-1R, ERK1/2, AKT, STAT3 serve as loading controls, as indicated. Data shown are the mean ± SEM of at least two independent experiments. Evaluation of luciferase activity in MCF-7 (**I**) and T47D (**J**) cells transiently transfected with a S100A7 (pS100A7) promoter construct and treated with IGF-1 (10 nM, 18 h) in the presence of OSI (10 μM), AG (25 μM), PD (300 nM), and LY (10 μM). Luciferase activity was normalized to the internal transfection control. Results are expressed as the % change of normalized RLU values relative to vehicle-treated cells. Data shown are the mean ± SEM of at least two independent experiments performed in triplicate. (*) *p* < 0.05; (**) *p* < 0.01; (***) *p* < 0.001.

Previously, ERK1/2 and AKT signaling cascades have emerged as pivotal transduction mediators involved in IGF1-dependent responses in cancer cells [6,23]. Along with these typical transducers, further intracellular mediators have been shown to bridge together IGF1-triggered signals with downstream effectors, including STAT3 [24,25]. Interestingly, S100A7 is a STAT3-regulated gene [17,26], thus suggesting that STAT3 could be engaged by IGF-1 toward the up-regulation of S100A7. In order to test this hypothesis, we first determined that IGF-1/IGF-1R induces the typical activation of the ERK1/2 and AKT cascades (Figure 4A–C). Interestingly, a clear activation of STAT3(Y705) was observed in both MCF-7 and T47D cells stimulated with IGF-1 (Figure 4D,E), however this effect was prevented in MCF-7 cells knock-out for IGF-1R via CRISPR-cas9 genome editing (Figure 4F). Previous data have demonstrated that ERK1/2, AKT, and STAT3 signaling are involved in the regulation of S100A7 expression in MCF-7 cells upon treatment with inflammatory cytokines [27]. On the basis of these data, we selectively inhibited each of the above kinases during IGF-1 treatment in order to test their involvement in the regulation of S100A7 expression by IGF-1. The inhibition of PI3K/AKT, ERK1/2 and STAT3 (Figure 4A–E) completely abrogated the S100A7 induction by IGF-1 in both MCF-7 and T47D cells (Figure 4G,H). The same pharmacological inhibitors were able to interfere with the transactivation of a S100A7 promoter (pS100A7) construct induced by IGF-1 (Figure 4I,J).

**Figure 5:**
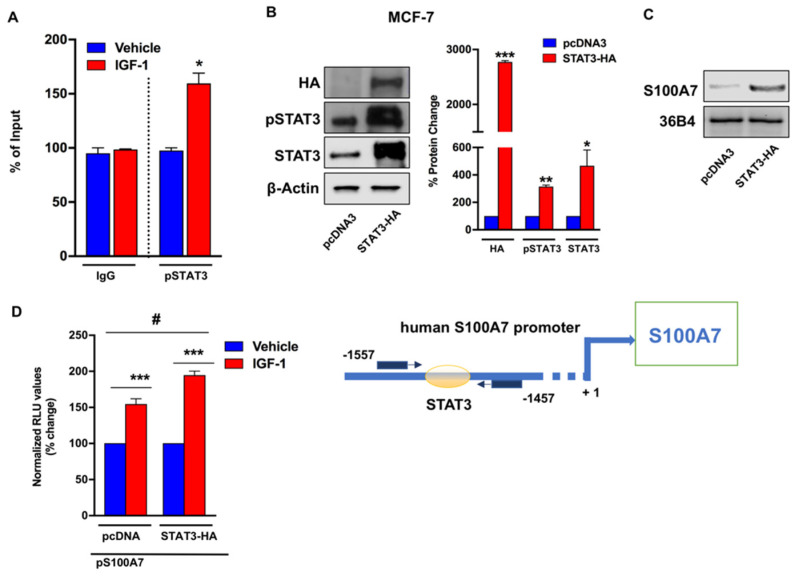
STAT3 is involved in the transcriptional activation of S100A7 by IGF-1. (**A**) IGF-1 (10 nM, 24 h) induces the recruitment of STAT3 (Y705) to the STAT3 binding site located within the S100A7 promoter sequence, as ascertained by Chromatin Immunoprecipitation (ChIP)-RT-PCR assay performed in MCF-7 cells. In control samples, nonspecific IgG was used instead of the primary antibody. Data shown are the mean ± SEM of at least two independent experiments performed in duplicate. (**B**) Representative immunoblots of HA, pSTAT3 (Y705), and STAT3 in MCF-7 cells which were transiently transfected with pcDNA3 and STAT3-HA for 48 h, to generate a constitutively activated STAT3 system. (**C**) Evaluation of S100A7 mRNA expression by RT-PCR in MCF-7 cells transfected with pcDNA3 and STAT3-HA for 48 h, as indicated. 36B4 gene serves as normalizing control. (**D**) The transactivation of a S100A7 (pS100A7) promoter plasmid observed after treatment with IGF-1 (10 nM, 18 h) is enhanced in MCF-7 cells transfected for 48 h with STAT3-HA, compared with cells transfected with pcDNA3 control vector. Results are expressed as the % change of normalized RLU values relative to vehicle-treated cells. Data shown represent the mean ± SEM of at least two independent experiments performed in triplicate. (*), (#) *p* < 0.05; (**) *p* < 0.01; (***) *p* < 0.001. Side panel shows a schematic representation of human S100A7 promoter carrying the STAT3-responsive site.

Next, chromatin immunoprecipitation assays performed in MCF-7 cells treated with IGF-1 revealed that activated STAT3(Y705) is recruited to a responsive site located within the human S100A7 promoter (Figure 5A). To confirm the role of STAT3 in S100A7 expression, we used a constitutively activated STAT3 system employing the plasmid pcDNA3-STAT3-HA (STAT3-HA), which contains the coding region of the STAT3 gene cloned into pcDNA3-HA vector. As shown in Figure 5B, transfection of the STAT3-HA plasmid in MCF-7 cells determined almost a 3-fold increase of STAT3 phosphorylation compared with cells transfected with pcDNA3 control vector. In these experimental conditions, the expression of S100A7 was similarly increased (Figure 5C), thus reinforcing the role of STAT3 activation in the regulation of S100A7. Likewise, the transactivation of a S100A7 promoter construct (pS100A7) induced by IGF-1 was enhanced in STAT3-overexpressing MCF-7 cells respect to cells transfected with an empty vector (pcDNA3) (Figure 5D). Together, these data suggest that IGF-1/IGF-1R axis triggers the activation of signaling cascades toward the STAT3-dependent regulation of S100A7.

### 2.3. IGF-1 Triggers Angiocrine Actions through the Involvement of S100A7/RAGE Signaling

**Figure 6:**
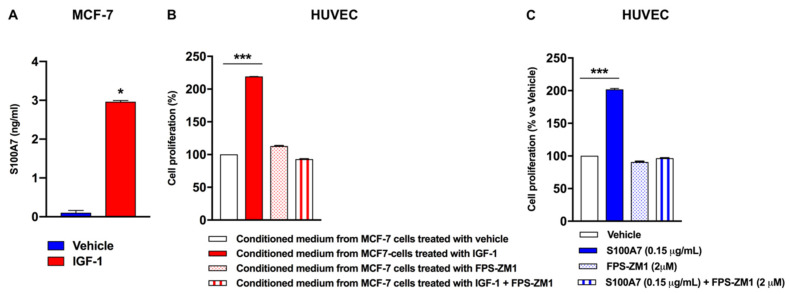
Paracrine release of S100A7 by BC cells induces proliferative effects in HUVECs. (**A**) Evaluation of S100A7 concentration in conditioned medium from MCF-7 cells treated with vehicle or IGF-1 (10 nM, 48 h), as evaluated by ELISA. Data shown are the mean ± SEM of at least three independent experiments performed in duplicate. (**B**) HUVECs were cultured in medium collected from MCF-7 cells treated with either vehicle or IGF-1 (10 nM, 48 h), in the presence of the RAGE inhibitor FPS-ZM1 (2 μM). Thereafter, HUVECs were subjected to SRB assay for the evaluation of cell growth. (**C**) HUVECs were cultured in 0.1% FBS in the presence of vehicle and human recombinant S100A7 (0.15μg/mL), alone and in combination with the RAGE inhibitor FPS-ZM1 (2 μM), as indicated. After 48 h, HUVECs were subjected to SRB assay for the evaluation of cell growth. Data shown are the mean ± SEM of at least 2 independent experiments performed in sextuplicate. (*) *p* < 0.05; (***) *p* < 0.001.

Previous studies have demonstrated that S100A7 binding to its receptor, namely RAGE, elicits angiogenic actions through multiple mechanisms including the activation of human vascular endothelial cells toward the acquisition of pro-angiogenic phenotype [14,28,29]. In this regard, it should be mentioned that endothelial cell proliferation is the main step involved in the angiogenic switch [30]. On the basis of these observations, we set out to determine whether S100A7 may trigger paracrine effects in adjacent endothelial cells, which express the S100A7 receptor namely RAGE (Figure S1C,D) [31]. First, we determined that in MCF-7 cells IGF-1 induces nearly a 3-fold increase in the amount of S100A7 released in cell medium, as assessed by ELISA (Figure 6A). Next, conditioned medium obtained from MCF-7 cells was collected and used as culture medium in human vascular endothelial cells (HUVECs), in order to evaluate HUVEC proliferation. We found that conditioned medium collected from IGF1-stimulated MCF-7 cells induced the proliferation of HUVECs, while this effect was prevented by adding the RAGE inhibitor FPS-ZM1 [32] to the conditioned medium (Figure 6B). Similar effects were observed in HUVECs cultured with human recombinant (hr) S100A7 in the presence of FPS-ZM1 (Figure 6C).

**Figure 7:**
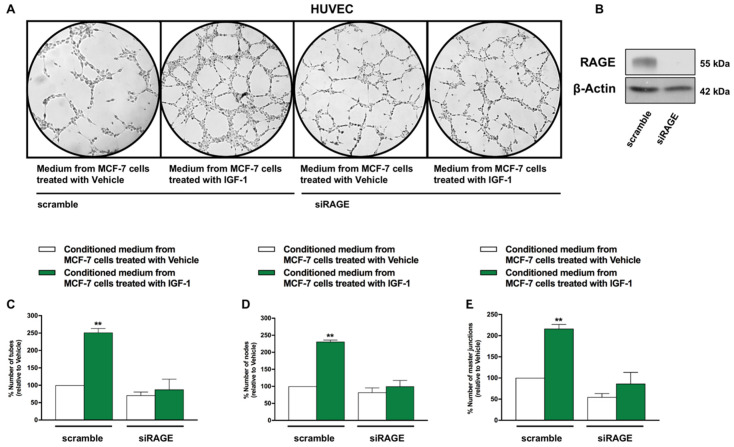
IGF-1 triggers the formation of endothelial tubes by activating the S100A7/RAGE signaling. (**A**) HUVECs were transfected with siRAGE (10 nM) or non-targeting scramble siRNA for 24 h. Thereafter, tube formation assay was performed on HUVECs, which were detached and cultured in medium collected from MCF-7 cells treated with either vehicle or IGF-1 (10 nM, 48 h). Tube formation was evaluated 8 h after HUVEC seeding. (**B**) Efficacy of RAGE silencing in HUVECs. (**C**–**E**) Quantification of the number of tubes, number of nodes and number of master junctions observed in HUVECs, as indicated. Data shown are the mean ± SEM of at least two independent experiments performed in duplicate. (**) *p* < 0.01.

Subsequently, the angiogenic response to the conditioned medium obtained from MCF-7 cells was assessed through endothelial cell tube formation assay [33]. As shown in Figure 7, we detected a ramified network of tubules in HUVECs cultured in conditioned medium collected from MCF-7 cells treated with IGF-1 (Figure 7A,B). This effect was no longer evident in HUVECs previously transfected with siRNA sequences specifically targeting RAGE (Figure 7A–E). Similar results were obtained performing tube formation assay using HUVECs cultured in medium collected from T47D cells treated with IGF-1 and silenced for IGF-1R expression (data not shown). Further corroborating these findings, the efficiency of endothelial tube formation was significantly reduced when HUVECs were cultured in conditioned medium from MCF-7 cells stimulated with IGF-1 in the presence of the RAGE inhibitor FPS-ZM1 (Figure S2A–C).

**Figure 8:**
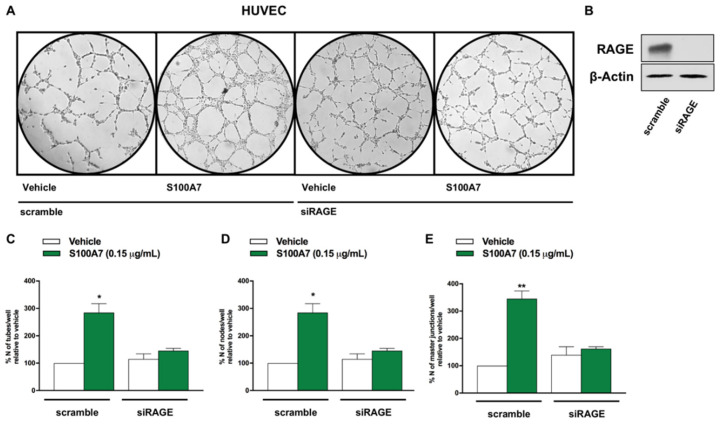
S100A7 triggers endothelial tube formation through the involvement of RAGE. (**A**) HUVECs were transfected with siRAGE (10 nM) or non-targeting scramble siRNA for 24 h. Thereafter, tube formation assay was performed on HUVECs, which were detached and cultured in Endothelial Basal Medium supplemented with either vehicle or S100A7 (0.15 μg/mL). Tube formation was evaluated 8 h after HUVEC seeding. (**B**) Efficacy of RAGE silencing in HUVECs. (**C**–**E**) Quantification of the number of tubes, number of nodes and number of master junctions observed in HUVECs, as indicated. Data shown are the mean ± SEM of at least two independent experiments performed in duplicate. (*) *p* < 0.05; (**) *p* < 0.01.

Additionally, increased endothelial tube formation was observed when HUVECs were directly stimulated with hrS100A7 (Figure 8), whereas this effect was prevented by RAGE silencing (Figure 8), as well as by the RAGE antagonist FPS-ZM1 (Figure S2D,E).

**Figure 9:**
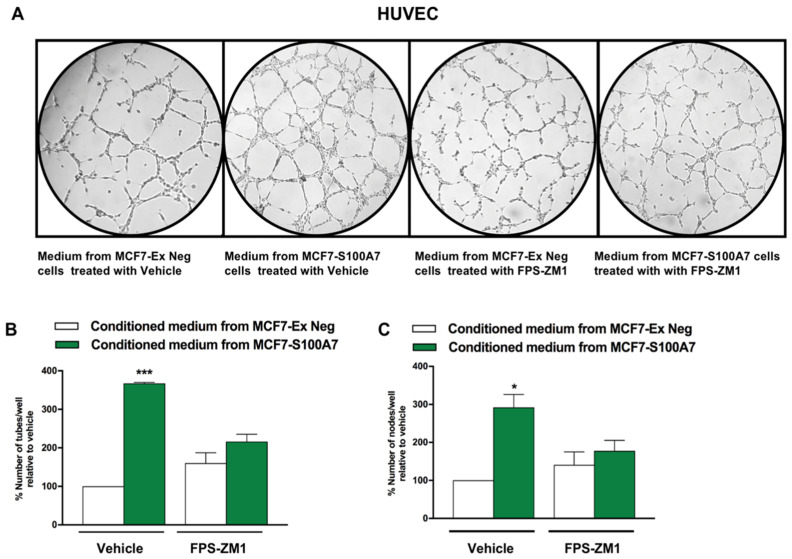
S100A7/RAGE signaling prompts endothelial tube formation. (**A**) Tube formation assay was performed in HUVECs which were cultured in medium collected from MCF7-Ex Neg (control) and MCF7-S100A7 (overexpressing S100A7) cells, in the presence of the RAGE inhibitor FPS-ZM1 (2 μM). Tube formation was evaluated 8 h after HUVEC seeding. (**B**,**C**) Quantification of the number of tubes and number of nodes observed in HUVECs, as indicated. Data shown are the mean ± SEM of at least two independent experiments performed in duplicate. (*) *p* < 0.05; (***) *p* < 0.001.

Last, we generated an isogenic MCF-7 cell line overexpressing human S100A7 via lentiviral transduction (Figure S1E,F). Interestingly, we observed a higher tube formation capacity in HUVECs cultured with conditioned medium obtained from MCF-7 cells transduced with S100A7 (MCF7-S100A7), compared with HUVECs cultured in medium obtained from MCF-7 cells transduced with an empty vector (MCF-7 Ex-Neg) (Figure 9). Worthy, this stimulatory effect was inhibited when the RAGE inhibitor FPS-ZM1 was added to the conditioned medium collected from MCF7-S100A7 cells (Figure 9). Altogether, these findings suggest that the IGF-1/IGF-1R axis primes the breast tumor microenvironment toward the acquisition of an angiogenic phenotype through the S100A7/RAGE signaling.

## 3. Discussion

In the current study, we investigated the role of the IGF-1/IGF-1R transduction pathway on the expression of the angiogenic protein S100A7 in BC. We established that in ER-positive BCs, S100A7 correlates with worse prognostic parameters and with higher tumor grade. By using cell models recapitulating the features of ER-positive BCs, we found that IGF-1 triggers the STAT3-dependent transcriptional activation of the S100A7 gene through IGF-1R. We characterized certain biological actions that might be elicited by S100A7 in the tumor microenvironment, with particular focus on its ability to promote breast tumor angiogenesis, as the angiogenic role of S100A7 is well acknowledged [14,28,29]. In particular, we found that the S100A7/RAGE axis is engaged in human vascular endothelial cells toward angiogenic responses, as demonstrated using HUVEC cultured in conditioned medium collected from IGF1-primed BC cells. Therefore, S100A7 may represent a novel effector through which the IGF-1 system executes molecular programs that modulate the breast tumor microenvironment toward the acquisition of negative features (as depicted in the graphical abstract). The IGF-1/IGF-1R pathway has a prominent oncogenic role in breast tumors, where it triggers direct autocrine proliferative and migratory actions on epithelial cancer cells [7,34,35]. In addition, the IGF-1/IGF-1R transduction pathway has been shown to coordinate a hub of molecular and biological interactions that facilitate the reciprocal cross-talk between BC cells and microenvironmental components toward the disease progression [6,36]. In this cotext, our study highlights that S100A7 may be considered as a novel angiogenic paracrine mediator of the IGF-1/IGF-1R action elicited in breast tumors.

The multi-gene family of the S100 proteins is a heterogeneous group of 21 known low molecular weight proteins, which display an elevated degree of structural similarity but distinct biological functions [37]. Typically, the S100 proteins exist as symmetric homodimers, with each subunit containing two Ca2+-binding/EF hand domains with helix-loop-helix conformation. Beyond serving as intracellular Ca2+ sensors, S100 proteins function as cytokine-like extracellular factors, thereby impacting on a number of biological functions [37]. Dysregulated expression of S100 proteins may occur in human cancers, therefore their use for predictive and prognostic purposes has been proposed [38]. The protein S100A7, also named psoriasin, was firstly identified as the most highly secreted protein in abnormally differentiated keratinocytes from psoriatic skin lesions, where S100A7 propagate aberrant immune and inflammatory signals [39,40]. Beyond contributing to the regulation of inflammation and innate immunity in several physio-pathological conditions, S100A7 has been shown to actively contribute to the progression of several neoplastic diseases, including BC [8]. Indeed, S100A7 has been shown to prime epithelial BC cells for enhanced proliferative and migratory capacity [13,27]. In addition, S100A7 may rearrange the BC microenvironment favoring the angiogenic and metastatic potential [11,12,14,16]. Most of these stimulatory actions have been demonstrated for ER-negative BCs, which exhibit elevated expression of S100A7 compared to ER-positive BCs [16,21,41]. Our analysis of the METABRIC dataset is in accordance with these findings, and support the concept that ER-negative BCs generally express higher levels of S100A7. Moreover, interrogating the same dataset, we found that elevated S100A7 levels correlate with higher tumor grade and worse outcome in patients with ER-positive BCs. Our data are in line with recently published studies indicating that S100A7 immunoreactivity is associated with worse prognostic factors, including distant metastasis-free interval and breast cancer-specific survival in ER-positive stage I-III BCs [42]. In addition, our analyses of in silico data are in accordance with previous findings showing that S100A7 expression is higher among patients who died of the disease or developed metastases [43]. Together, these observations convincingly support the idea that S100A7 expression tends to progressively increase with the transition of the disease from a locally in-situ lesion to a pre-invasive and more aggressive phenotype in ER-positive BCs. Still open questions remain on the differential expression of S100A7 observed in ER-positive vs ER-negative BCs. This is a general feature shared by all members belonging to the gene of the S100 family, as evidenced by in silico multiomics analysis in BC [44]. Such observation would suggest the existence of a shared mechanism, yet to be identified, of gene inactivation for the S100 proteins in ER-positive BCs. Nevertheless, the transcription of S100 genes in ER-positive BCs can be immediately boosted in response to certain stimuli. For instance, in ER-positive MCF-7 cells, estrogens induce the expression of S100A7 through diverse mechanisms, including the activation of ER, as well as the inhibition of gene methylation [9,10,45]. Therefore, it could be assumed that multiple events such as epigenetic mechanisms and differentiation-related dynamics may be involved in the differential expression of S100A7 in ER-positive vs ER-negative BCs.

Despite our in-silico analysis of the METABRIC dataset did not provide a positive correlation between the expression of S100A7 and IGF/IGF-1R in the examined cohort of patients (data not shown), such discrepancy could be due to intrinsic limits of the gene expression methodologies used, as well as to the relative contribution of the multiple cellular component that constitute the breast tumor samples (cancer associated fibroblasts, macrophages, adipocytes, immune and endothelial cells). In fact, S100A7 regulation occurs in a cell-type specific manner, while the data collected in the METABRIC cohort are representative of the whole tumor tissue.

S100A7 could be regarded as an early orchestrator of the metastatic switch for its ability to establish a propaedeutic vascular niche through the manipulation of the tumor microenvironment. In this context, chromosomal copy-number amplification at 1q21.3, a locus encoding for S100A family members like S100A7, associates with BC recurrence, which is strictly related with stem features and metastatic potential [46].

The up-regulation of S100A7 in BC cells may occur as a consequence of detrimental stimuli involved in disease malignancy, including inflammatory cytokines and growth factors as EGF [11,27]. In this context, our study includes IGF-1 among the factors implicated in S100A7 up-regulation in BCs.

IGF-1 typically signals through IGF-1R and two main transduction pathways: the PI3K/AKT and the Ras/MAPK signaling cascades [23,47]. In addition, the IGF-1/IGF-1R axis signals also through the Janus kinase/signal transducer and activator of transcription pathway (JAK/STAT) pathway [24,25]. Altogether, these intracellular kinases engage transcription factors toward the regulation of IGF-1R target genes. Accordingly, here we show that IGF-1 through IGF-1R triggers the activation of ERK1/2, AKT and STAT3, which are involved in the up-regulation of S100A7 protein expression, as evidenced by gene silencing and pharmacological inhibitors. On the basis of our findings these transduction pathways could converge on S100A7, however the relative contribution of each signaling route deserves further investigation. Anyway, our data nicely fit with previous investigations showing that in MCF-7 and T47D BC cells, inflammatory cytokines like Oncostatin-M and IL-6 prompt the increase of S100A7 through the involvement of ERK1/2, PI3K/AKT and STAT3 signaling pathways [27]. In the aforementioned study, the authors showed that the blockade of both STAT3 phosphorylation by the use of a STAT3 Y705F mutant construct as well as STAT3 transactivation capacity are implicated in the up-regulation of S100A7 by Oncostatin-M and IL-6 in BC cells (27), thus corroborating the direct and pivotal role played by STAT3 in the regulation of S100A7 expression. Worthy, STAT3 may directly transactivate the S100A7 promoter in normal human keratinocytes [48], corroborating our findings on the recruitment of phosphorylated STAT3 on the human S100A7 promoter following stimulation with IGF-1. Likewise, we have demonstrated that constitutive activation of STAT3 is sufficient to increase S100A7 levels, thus supporting the involvement of this transcription factor in the regulation of S100A7.

Nevertheless, additional details on the molecular mechanisms involved in the activation of STAT3 by IGF-1 and the STAT3-dependent transcription of S100A7 could be retrieved in further studies aimed at interrogating whether IGF-1 differentially regulates STAT3 nuclear translocation, and whether IGF-1 affects both STAT3 phosphorylation and DNA-binding capability.

The paracrine release of S100A7 into the tumor milieu indicates that this protein enacts biological programs dictated by signal originating in the primary tumor to educate surrounding endothelial cells toward an activated angiogenic phenotype. By facilitating the communications between BC cells and adjacent endothelial cells, S100A7 may be included among the soluble mediators of the microenvironmental secretome, which has gained enormous research interest for providing a valid platform in novel anti-cancer strategies [49]. Once released in the extracellular environment, S100A7 induces biological responses by binding to its receptor RAGE, which is a multi-ligand receptor implicated in the regulation of innate immunity, as well as in the pathogenesis of several inflammatory conditions [31]. Initially designated as a decoy receptor for the Advanced Glycation End Products generated during hyperglycemic states, RAGE has currently been acknowledged as a signaling receptor for a number of ligands, including S100A7 [31]. RAGE pathway is activated in conditions of de-regulated IGF/Insulin signaling and participates to the establishment of low-grade chronic inflammation and insulin resistance [20,50]. The S100A7/RAGE pathway has been shown to support neoplastic progression in diverse human tumors, including BC. In this regard, Nasser and collaborators using a transgenic mouse model of BC determined that S100A7 drives metastatic evolution through RAGE [12]. Worthy, in our model system the inhibition of RAGE was sufficient to block S100A7-induced angiogenic response, thus suggesting that blocking the S100A7/RAGE pathway could be considered a feasible option in order to halt aberrant angiogenesis. Altogether our results indicate that S100A7 is a novel stimulatory target of the IGF-1 action in BC. Bridging together signals generated at the cross-road between BC cells and the intra-tumor vascular niche, S100A7 may be regarded as a novel therapeutic target for a clear-cut manipulation of the tumor microenvironment toward better clinical outcomes of BC patients, especially when affected by disorders associated with de-regulated IGF-1/IGF-1R pathway.

## 4. Materials and Methods

### 4.1. Reagents

Insulin-like growth factor 1 (IGF-1) was from Prepotech. FPS-ZM-1 was purchased from Sigma-Aldrich (distributed by Biogenerica); AG490 was from Calbiochem. Linsitinib (OSI-906), PD0325901, and LY294002 were purchased from Selleck Chemicals. Human recombinant (hr) S100A7 protein was from R&D systems. All compounds were solubilized in DMSO, except for IGF-1 and S100A7, which were dissolved in 10 mM Acetic Acid and PBS, respectively. 

### 4.2. Publicly Available Molecular Datasets

Bioinformatics analyses were performed using the multiomic publicly available dataset Molecular Taxonomy of Breast Cancer International Consortium (METABRIC) [22]. The clinical information and the microarray gene expression data (Log2 transformed intensity values) of the METABRIC cohort were retrieved from cBioPortal for Cancer Genomics (http://www.cbioportal.org/, accessed on 12 December 2020) on November 16th 2020. Samples (n. 2509) were classified on the basis of the estrogen receptor (ER) expression levels, as assessed by immunohistochemistry. Gene expression data and clinical information were filtered for missing values, the final filtering resulted in 1459 ER-positive BC from a total amount of 1904 samples. All bioinformatics analyses were carried out using R Studio and the box-plots were generated with the tydiverse package.

### 4.3. Survival Analysis

The overall survival (OS) of ER-positive BC patients was ascertained using METABRIC S100A7 gene expression data. The samples were filtered for missing values as well as the vital status. In particular, patients classified as “died of other causes” were excluded. The survivALL package was employed to examine Cox proportional hazards for all possible points-of-separation (low-high cut-points), the cut-point with the lowest p-value [51] was used in order to separate the patients into the high (*n* = 162) and low (*n* = 785) S100A7 expression levels. The Kaplan–Meier survival curve was generated using the survival and the survminer packages.

### 4.4. Cell Cultures

The MCF-7 and T47D breast cancer cells, obtained from ATCC, were maintained in MEM and high glucose DMEM, respectively (Sigma-Aldrich, Merck, Milan, Italy) supplemented with 10% fetal bovine serum (FBS), 1% penicillin/streptomycin and 1% Glutamine (Thermo Fisher Scientific, Life Technology, Milan, Italy). Human umbilical vein endothelial cells (HUVECs) were obtained from ATCC and cultured in Endothelial Growth Medium (EGM) (Lonza), supplemented with 2% FBS (Lonza). 293Ta packaging cells, obtained from Genecopoeia, were maintained in high glucose DMEM supplemented with 10% fetal bovine serum (FBS), 1% penicillin/streptomycin and 1% Glutamax. MCF-7 Clustered Regularly Interspaced Short Palindromic Repeats (CRISPR)-Cas9, knock-out (KO)-IGF1R were purchased from Applied Biological Materials (Richmond, BC, Canada). MCF7-Ex Neg cells and MCF7-S100A7 cells, which overexpress the human S100A7 protein, were generated by lentiviral transduction. All cell lines were grown in a 37 °C incubator with 5% CO2. Cells were switched to 1% charcoal-stripped (CT) serum the day before the experiment.

### 4.5. Lentiviral Gene Transduction

A stable S100A7-overexpressing MCF-7 cell line (MCF7-S100A7) was generated by lentiviral gene transduction. Lentiviral plasmids, packaging cells and reagents were from Genecopoeia. Forty-eight hours after seeding, 293Ta packaging cells were transfected with lentiviral vectors encoding for human S100A7 combined with the fluorescent tag mCherry (EX-A4051-Lv111 ORF expression clone for S100A7), or empty vector (EV, EX-NEG-Lv111 Empty control vector for pReceiver Lv111), using the Lenti-PacTM HIV Expression Packaging Kit, according to the manufacturer’s instructions. Two days post-transfection, lentivirus-containing culture medium was passed through a 0.45 μm filter and added to the target cells (MCF-7 cells) in the presence of 5μg/mL polybrene. Transduced MCF-7 cells were selected with 2.5 μg/mL puromycin.

### 4.6. Gene Silencing and Plasmids

For knocking down IGF-1R expression, cells were seeded in six-well multi-dishes and transiently transfected the consecutive day at 70% confluence using 10 nM small interfering RNAs (siRNA) smart pool, which consists of 4 siRNA sequences, (Dharmacon, distributed by Biogenerica, Catania, Italy) or scramble non-targeting control with Lipofectamine RNAiMAX (Thermo Fisher, Life Technology, Milan, Italy) for 24 h, prior to treatments. For knocking down RAGE expression, siTran 1.0 (Origene, distributed by Tema Ricerca, Bologna, Italy), was mixed with two siRAGE targeting sequences or a non-targeting scramble control (Origene) (10 nM) for 24 h, prior to treatments. For the generation of a constitutively active STAT3 model system we used the plasmid STAT3-HA, a kind gift of Dr. Cheng (Taipei Medical University, Taipei, Taiwan) [26]. The plasmid STAT3-HA, which contains the coding region of the STAT3 gene cloned into pcDNA3-HA vector, was transfected in 70% confluent MCF-7 cells using X-treme GENE 9 DNA Transfection Reagent (Roche Diagnostics, Merck Life Science), as recommended by the manufacturer, for 24 h prior treatments.

### 4.7. Gene Reporter Assays

The human S100A7 promoter-luciferase construct containing full-length S100A7 promoter, identified as the 5′-upstream 1551-bp length of S100A7 sequence from the National Center for Biotechnology Information (NCBI) database, was a kind gift from Dr. Cheng (Taipei Medical University, Taipei, Taiwan) [26]. Transfections were performed using X-treme GENE 9 DNA transfection reagent as recommended by the manufacturer (Roche Diagnostics), with a mixture containing 0.5 µg of reporter plasmid and 10 ng of pRL-TK. After 24 h, cells were treated with IGF-1, as indicated. For co-transfection experiments, cells were previously transfected with 1 µg STAT3-HA in the presence of a mixture containing 0.5 µg of reporter plasmid and 10 ng of pRL-TK, using X-treme GENE 9 DNA Transfection reagent. After 24 h, cells were treated for 18 h with IGF-1 in 1% charcoal treated (CT) FBS. Luciferase activity was measured with the Dual Luciferase Kit (Promega) normalized to the internal transfection control provided by Renilla luciferase activity. The normalized relative light unit values were expressed as the average % change of luciferase activity relative to the vehicle-treated cells, whose luciferase activity was set as 100%.

### 4.8. Gene Expression Studies

Total RNA was extracted from cell cultures using the TRIzol commercial kit (Thermo Fisher Scientific) according to the manufacturer’s protocol. RNA was quantified spectrophotometrically, and quality was checked by electrophoresis through agarose gels stained with ethidium bromide. Only samples that were not degraded and showed clear 18 and 28 S bands under UV light were used for RT-PCR. Total cDNA was synthesized from the RNA by reverse transcription as previously described [18]. The expression of selected genes was quantified by real-time PCR using ABI 7500 Real-Time PCR System (Applied Biosystems) with probe, primer sets and SYBR Green chemistry. mRNA quantification was performed using the comparative cycle threshold (CT) method (DDCt). Primer sequences are as follows: hS100A7 Fwd: 5′-TGCATCTCCATCTTCTACCCAAGT-3′ and Rev: 5′-CCGACTGTGAGTGCCACTGT-3′; hRAGE Fwd: 5′-GGACCCTTAGCTGGCACTTAGA-3′; hRAGE Rv: 5′- GAGTCCCGTCTCAGGGTGTCT-3′; h36B4 Fwd: 5′- GGCGTCCCCCAACTTCTTA -3′ and Rev: 5′- GGGCATCACAGACCTGTTATT -3′. hGAPDH: Fwd: 5′-CAAGGCTGTGGGCAAGGT-3′ and Rev: 5′-GGAAGGCCATGCCAGTGA-3′. Assays were performed in duplicate, the results were normalized for 36B4 expression and then calculated as fold induction of RNA expression. For classic RT-PCR analysis, we used the kit Bioline PCR (Bioline, distributed by Biogenerica, Catania, Italy); amplification products were resolved on 2% agarose gel and visualized using Chemidoc Molecular Imager^®^ Gel Doc™ XR+ System with Image Lab™ Software (Bio-Rad, Hercules, CA, USA).

### 4.9. Western Blot Analysis

Cells were processed according to the previously described protocol [52] to obtain protein lysate that was electrophoresed through a reducing SDS 7.5, 10 or 15% (*w*/*v*) polyacrylamide gel, electroblotted onto a nitrocellulose membrane and probed with primary antibodies against S100A7 (36006), p-STAT3 (Y705), STAT3 (124H6), IGF-1R (3027S), pIGF-1R (Y1135/Y1136), ERK1/2 (9102S), pAKT (S473) D9E, AKT (C67E7), RAGE (D1A12), (all purchased from Cell Signaling Technology), pERK1/2 (E4), ERα (D-12) (Santa Cruz Biotechnology, DBA), and HA MMS-101R (Covance). Proteins were detected by horseradish peroxidase-linked secondary antibodies (Cell Signaling, distributed by Euroclone, Milan, Italy) and revealed using the West Pico Chemiluminescent Substrate (Thermo Fisher Scientific, Life Technology, Milan, Italy). Chemiluminescent signal was revealed on Amersham high performance chemiluminescence films (Hyperfilms Amersham, distributed by Biogenerica, Catania, Italy), or using the LI-COR Odyssey 2800 (Li-COR Inc., Lincoln, NE, USA) and the software ImageStudioLite (version 5.2). β-actin (Sigma Aldrich, Merck, Milan, Italy) served as loading control. Original western blots and densitometric analyses of all blots shown are reported in Figures S3–S8.

### 4.10. Chromatin Immunoprecipitation (ChIP) Assay

MCF-7 cells were grown in 10-cm dishes to 60 to 70% confluence, deprived in 1% charcoal stripped FBS (CT) and then treated with vehicle or 10 nM IGF-1 for 24 h. Thereafter, cells were cross-linked with 1% formaldehyde and sonicated. Supernatants were immuno-cleared with salmon DNA/protein A-agarose (Millipore) and immunoprecipitated with anti-pSTAT3 (Y705) antibody or nonspecific IgG (Cell Signaling Technology). Pellets were washed, eluted with a buffer consisting of 1% SDS and 0.1 mol/L NaHCO3, and digested with proteinase K (Sigma Aldrich). DNA was obtained by phenol/chloroform extractions and precipitated with ethanol. The yield of target region DNA in each sample after ChIP was analyzed by qRT-PCR. The primer pairs for the human S100A7 promoter containing the putative 100 bp STAT3 binding located on the human S100A7 promoter are as follows: 5’-GCTCTTTGTCCAAACACACACA-3’ (Fwd) and 5’-GGCACTTCTAGAAAACGCAAAG-3’ (Rv). Data were normalized to the input for the immunoprecipitation.

### 4.11. Conditioned Medium

MCF-7 cells were cultured in regular growth medium, then cells were washed twice with PBS and treated for 48 h with IGF-1 in charcoal treated (CT) FBS. Thereafter, the supernatants were collected and centrifuged at 3500 rpm for 5 min to remove cell debris. 15 mL of supernatant were collected and concentrated at 4000g for 30 min, using the centrifuge filters AMICON ULTRA 15mL NMWL (Millipore), as recommended by the manufacturer. The concentrated supernatant was used as conditioned medium in HUVECs.

### 4.12. ELISA

The concentration of S100A7 in cell supernatants, which were obtained as described above, was determined by enzyme linked immunosorbent assay (ELISA) kit (CircuLex™, cat# CY-8073, distributed by Voden, Rome, Italy) according to manufacturer’s instructions. Briefly, 100 μL of samples and standards (ranging from 0.12 to 90 ng/mL) were added to the wells. After incubation and washing, horseradish peroxidase (HRP) conjugated antibody was added, incubated and then wells were washed before the addition of substrate reagent. The reaction was stopped and the plate was read at 450 nm using a microplate-reader Wallac Victor 1420 (PerkinElmer, San Diego, CA, USA). The limit of detection was better than 0.12 ng/mL of sample.

### 4.13. Tube Formation Assay

The day before the experiment, confluent HUVECs were starved overnight at 37 °C in serum free medium. Cultrex Reduced Growth Factor Basement Membrane Matrix^®^ (R&D Systems, Tema Ricerca) was thawed overnight at 4 °C on ice, plated on the bottom of pre-chilled 96 well-plates and left at 37 °C for 1 h for gelification. Starved HUVECs were collected by enzymatic detachment (0.25% trypsin-EDTA solution, Thermo Fisher Scientific), counted and resuspended in conditioned medium from MCF-7 cells or in EBM medium (0.1% FBS) with or without treatments. Then, 10,000 cells/well were seeded on Matrigel and incubated at 37 °C. Tube formation was observed 8 h after cell seeding and quantified by using the software NIH ImageJ (National Institutes of Health (NIH), Rockville Pike, Bethesda, MD, USA).

### 4.14. Sulphorhodamine B (SRB) Assay

Protein content in viable HUVECs was assessed by using the sulphorhodamine (SRB) assay. After treatments, cells were fixed with 10% trichloroacetic acid (TCA) (Sigma Aldrich) for 1 h in the cold room, and dried overnight at room temperature. Then, cells were incubated with SRB (Sigma Aldrich, Merck, Milan, Italy) for 15 min, washed twice with 1% acetic acid, and air dried for at least 1 h. Finally, the protein-bound dye was dissolved in a 10 mM Tris pH 8.8 solution and read using the plate reader Wallac Victor 1420 (PerkinElmer, San Diego, CA, USA) at 540 nm. As SRB stechiometrically binds to proteins, the amount of bound dye was thereafter used as a proxy for cell mass and employed to extrapolate the rate of cell proliferation [53].

### 4.15. Statistical Analysis

Statistical analysis was performed using t-tests and Spearman correlations. *p* < 0.05 was considered statistically significant.

## 5. Conclusions

The present study holds significant potential from a translational standpoint. In fact, the 16–20% of BC patients are affected by metabolic disorders associated with aberrant IGF-1 signaling, like obesity and type-2 diabetes. Currently, no specific guidelines are available to the clinicians for the treatment of breast cancer patients with concomitant obesity and/or diabetes, both the diseases being managed separately. In these patients, the lack of coordination of care and the consequent potential drug interactions can lead to dispersion of health care resources, less effectiveness of treatment and higher mortality rates.

Therefore, the urge to better characterize from a molecular point of view the nodes of interactions that characterize the progression of breast disease in conditions of altered IGFs signaling. 

However, targeting the IGF-1/IGF-1R as an anticancer therapeutic option has provided poor benefit in clinical settings, due to the ability of the IGF-1 pathway to signal through multiple transduction mediators and alternate routes that may trigger stimulatory signals in response to IGF-1R blockade. Our results point at novel actionable effectors of the IGF-1/IGF-1R pathway. Indeed, the protein S100A7, which is regarded as an early orchestrator of the acquisition of invasive properties, elicits angiogenic actions through the binding to RAGE. This receptor, which is known to be actively implicated in the pathogenesis of inflammation in obesity, diabetes and cancer, is a druggable target whose inhibition may be achieved using a broad repertoire of small molecules, peptides, neutralizing antibodies, as well as by repurposing already available FDA approved drugs [54,55]. The evidence that IGF-1 triggers S100A7 up-regulation to drive the breast tumor microenvironment toward angiogenic programs suggests that we deepen our knowledge into the potential of the S100A7/RAGE pathway in breast cancer, particularly in those metabolic disorders characterized by enhanced activation of the IGF-1/IGF-1R pathway.

## Data Availability

The data presented in this study are available on request from the corresponding author. Publicly available datasets were analyzed in this study. This data can be found here: http://www.cbioportal.org/ (accessed on 12 December 2020).

## Supplementary Materials

The following are available online at https://www.mdpi.com/2072-6694/13/4/621/s1, Figures S1–S8. Figure S1: Expression of ERα, IGF-1R, RAGE and S100A7. Figure S2: Angiocrine effects of IGF-1 through the S100A7/RAGE pathway. Figure S3: Original Western blots and densitometric analyses relative to Figure 2. Figure S4: Original Western blots and densitometric analyses relative to Figure 3. Figure S5: Original Western blots and densitometric analyses relative to Figure 4. Figure S6: Original Western blots and densitometric analyses relative to Figure 5. Figure S7: Original Western blots and densitometric analyses relative to Figure 7 and Figure 8. Figure S8: Original Western blots and densitometric analyses relative to Figure S1.
