# Supplementary material for: Activation of the S100A7/RAGE Pathway by IGF-1 Contributes to Angiogenesis in Breast Cancer
Source: Cancers (Basel). 2021 Feb 4;13(4):621. doi: 10.3390/cancers13040621 (PMC7915817; doi:10.3390/cancers13040621)
Supplement: Supplementary file 1 [file cancers-13-00621-s001.pdf]

cancers MDPI

Supplementary Materials

Activation of the S100A7/RAGE Pathway by IGF-1 Contributes to Angiogenesis in Breast Cancer

Maria Grazia Muoio [1,2], Marianna Talia [2], Rosamaria Lappano [2], Andrew H. Sims [3], Veronica Vella [1], Francesca Cirillo [2], Livia Manzella [4,5], Marika Giuliano [1], Marcello Maggiolini [2], Antonino Belfiore [1,*] and Ernestina Marianna De Francesco [1,*]

SUPPLEMENTARY INFORMATION

Supplementary Figures

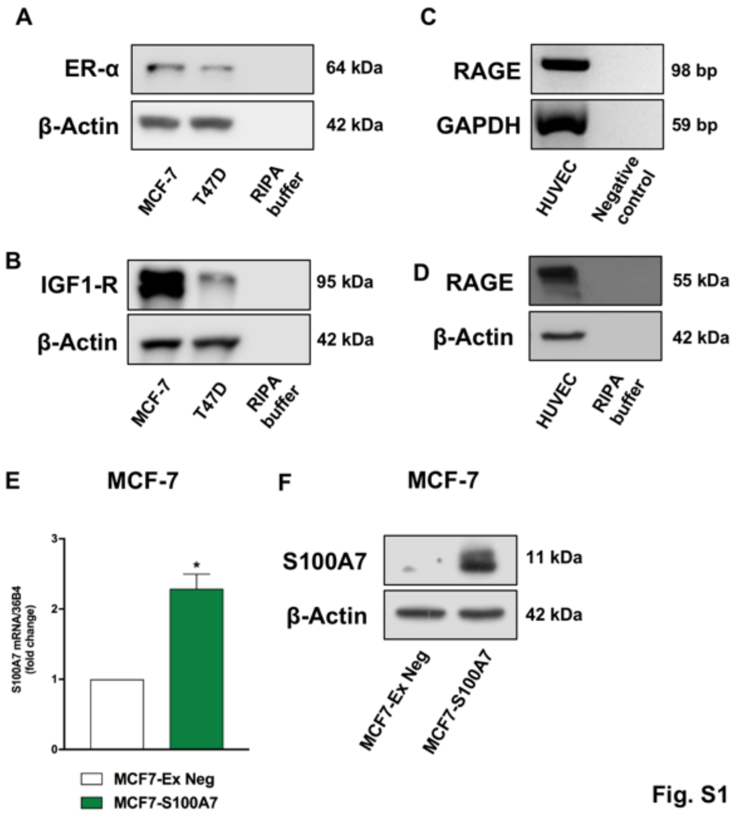

Figure S1. Expression of ERα, IGF-1R, RAGE and S100A7. **(A-B)** Evaluation of ERα and IGF-1R protein expression by western blotting in MCF-7 and T47D cells. Lysis buffer without proteins was used in lane 3. β-actin serves as loading control. Evaluation of RAGE expression in HUVECs by RT-PCR (**C**) and western blotting (**D**), as indicated. RNase free water and protein lysis buffer were used as negative controls in PCR and western blotting experiments, respectively. Efficacy of S100A7 overexpression obtained by lentiviral transduction in MCF7-Ex Neg (control) and MCF7-S100A7 (overexpressing S100A7) cells, as evaluated by qRT-PCR (**E**) and western blotting (**F**).

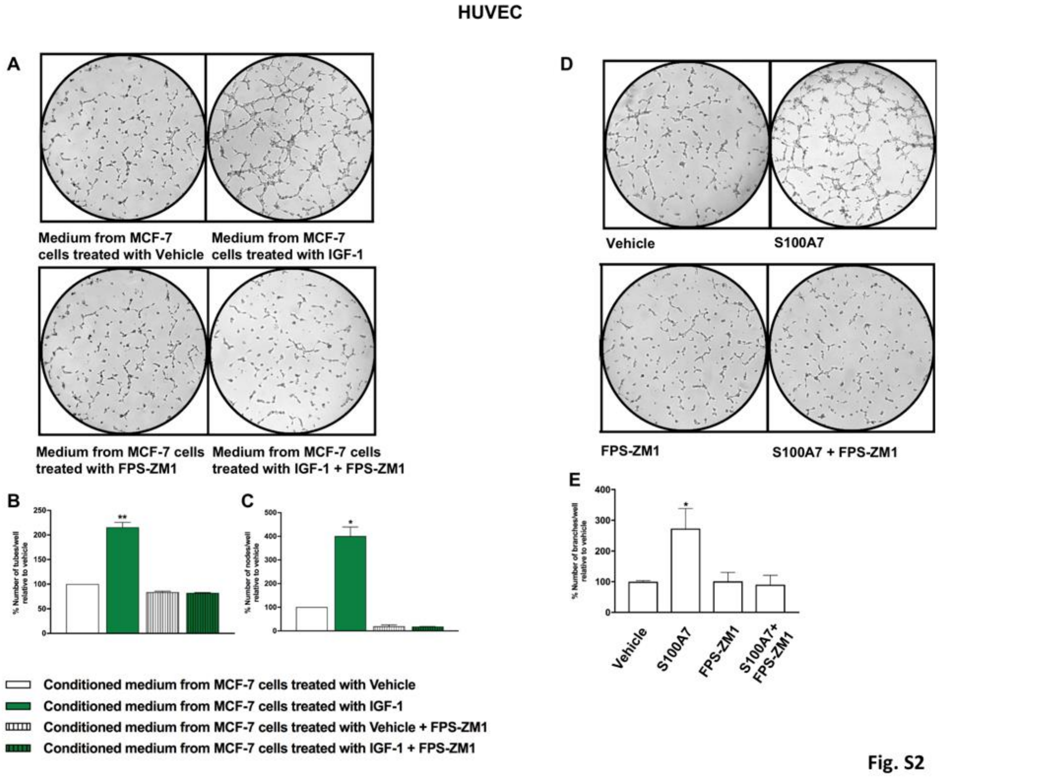

Figure S2. Angiocrine effects of IGF-1 through the S100A7/RAGE pathway. (**A**) Tube formation assay was performed in HUVECs cultured in conditioned medium collected from MCF-7 cells, which were treated with vehicle or IGF-1 (10 nM, 48 hours). The RAGE inhibitor FPS-ZM1 (2 μM) was added to the conditioned medium at the moment of HUVEC seeding. (**B-C**) Quantification of the number of tubes and number of nodes observed in HUVECs, as indicated. (**D**) Tube formation assay was performed on HUVECs, which were treated with vehicle or S100A7 (0.15μg/ml), alone and in combination with the RAGE inhibitor FPS-ZM1 (2 μM). Tube formation was evaluated 8 hours after HUVEC seeding. (**E**) Quantification of the number of branches in HUVECs, as indicated. Data shown are the mean ± SEM of at least two independent experiments performed in duplicate. (*) $p < 0.05$; (**) $p < 0.01$.

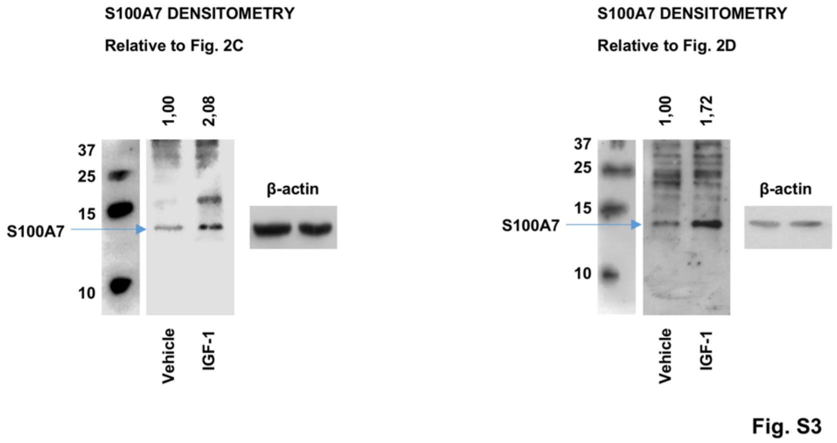

Figure S3. Original Western blots and densitometric analyses relative to Figure 2. The figure shows the original Western blots contained in Figure 2, as indicated, as well as the corresponding densitometric analyses.

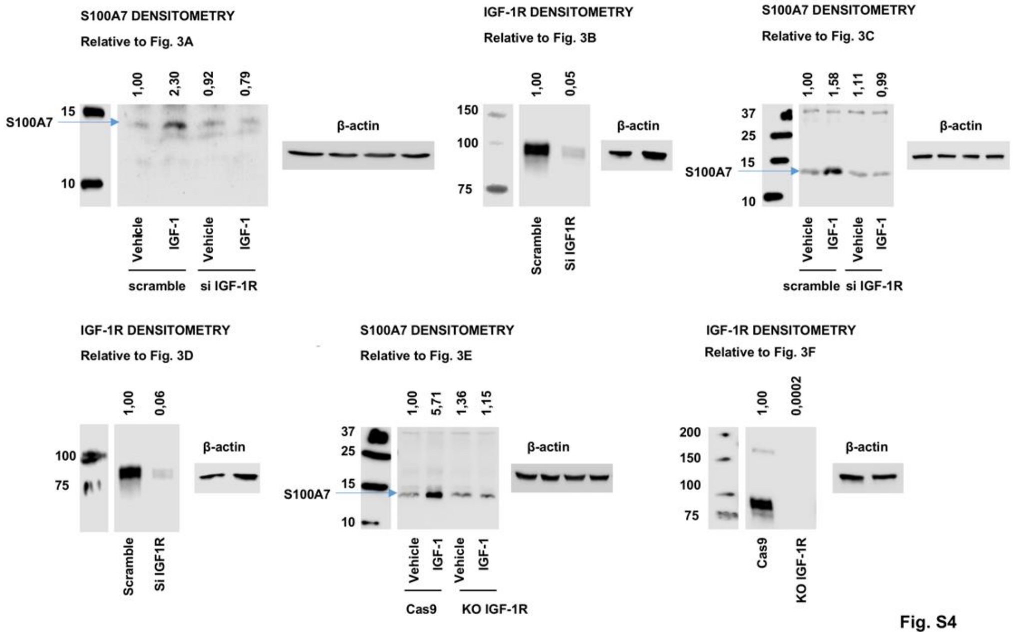

Figure S4. Original Western blots and densitometric analyses relative to Figure 3. The figure shows the original Western blots contained in Figure 3, as indicated, as well as the corresponding densitometric analyses.

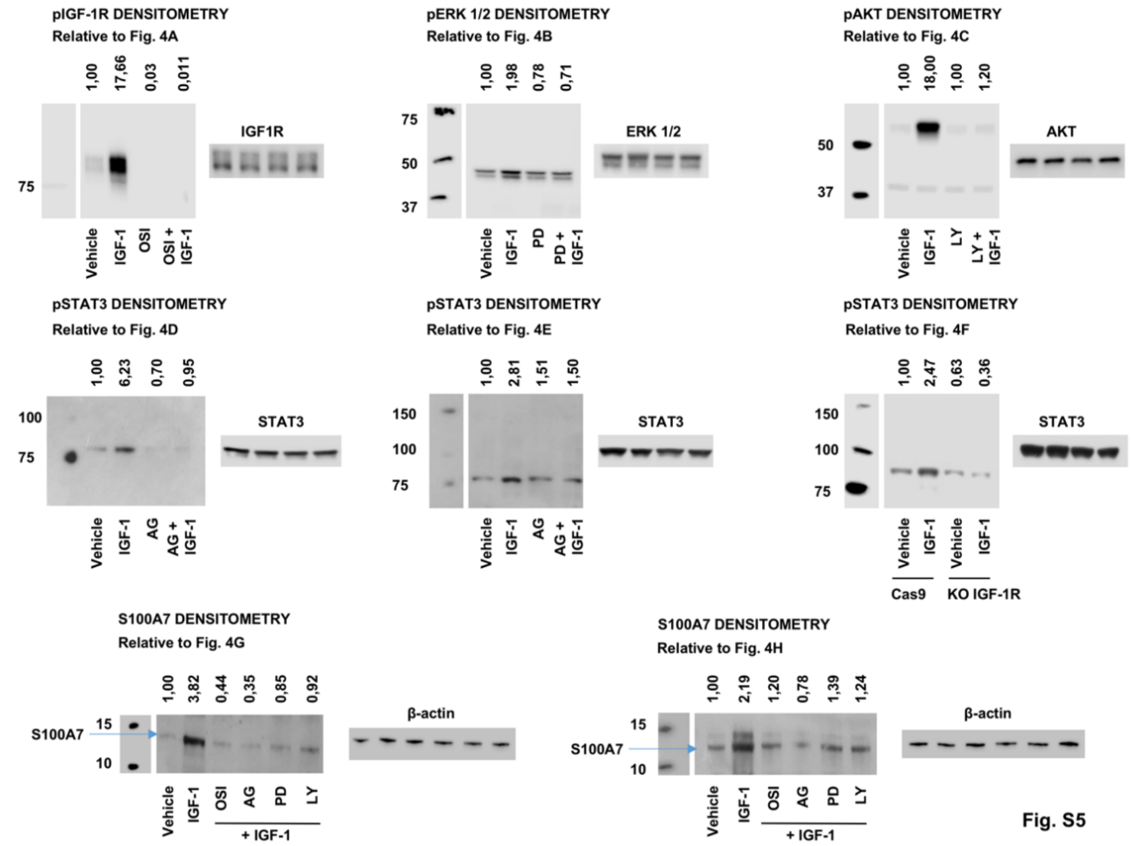

Figure S5. Original Western blots and densitometric analyses relative to Figure 4. The figure shows the original Western blots contained in Figure 4, as indicated, as well as the corresponding densitometric analyses.

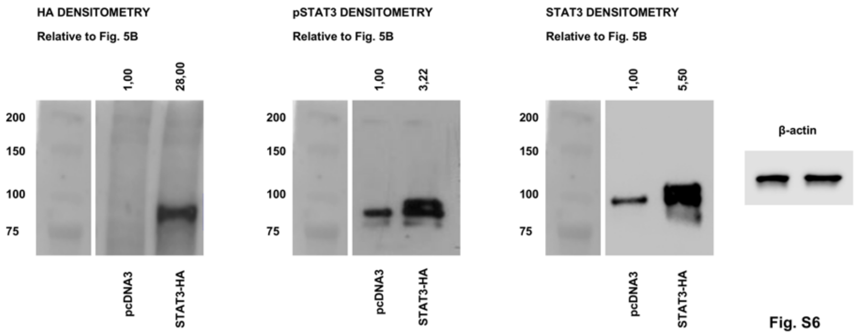

Figure S6. Original Western blots and densitometric analyses relative to Figure 5. The figure shows the original Western blots contained in Figure 5, as indicated, as well as the corresponding densitometric analyses.

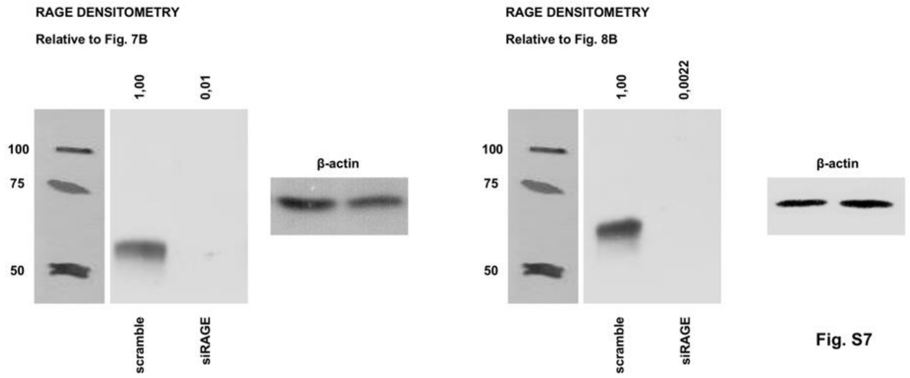

Figure S7. Original Western blots and densitometric analyses relative to Figure 7 and Figure 8. The figure shows the original Western blots contained in Figure 7 and Figure 8, as indicated, as well as the corresponding densitometric analyses.

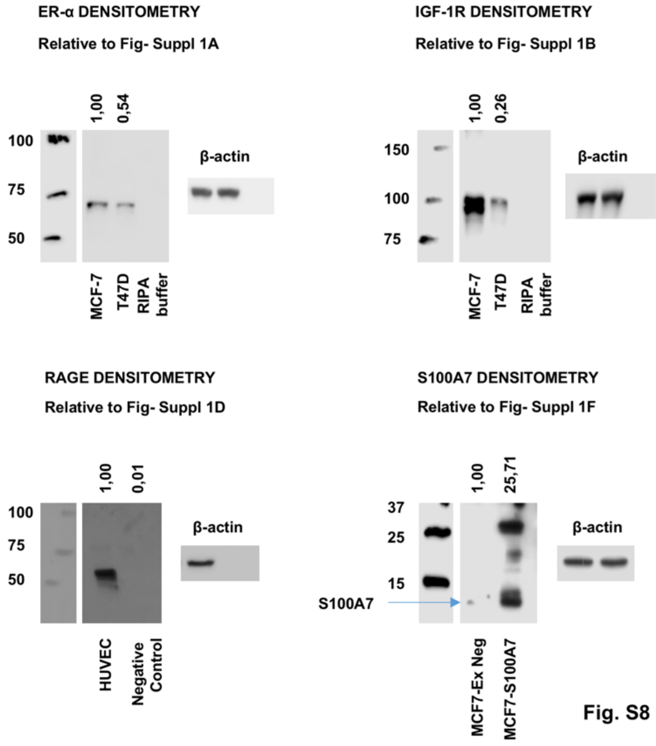

Figure S8. Original Western blots and densitometric analyses relative to Figure S1. The figure shows the original Western blots contained in Figure S1, as indicated, as well as the corresponding densitometric analyses.
